# Circulating unmethylated insulin DNA as a potential non-invasive biomarker of beta cell death in type 1 Diabetes: a review and future prospect

**DOI:** 10.1186/s13148-017-0343-5

**Published:** 2017-04-26

**Authors:** Kuo Zhang, Guigao Lin, Yanxi Han, Jiehong Xie, Jinming Li

**Affiliations:** National Center for Clinical Laboratories, Beijing Hospital, National Center of Gerontology, No.1 Dahua Road, Dong Dan, Beijing, 100730 People’s Republic of China

**Keywords:** Type 1 diabetes, Early detection, Cell-free DNA, Unmethylated insulin DNA, Molecular biomarkers

## Abstract

**Background:**

The early detection of type 1 diabetes (T1D) largely depends on a reliable approach to monitor β cell loss. An effective way to evaluate the decline of β cell mass would allow early preventative intervention to preserve insulin secretion.

**Main body:**

Recent progress in the development of novel biomarkers, based on tissue-specific methylation patterns, has inspired relevant studies in T1D. In this review, we focus on the application of circulating β cell-derived unmethylated insulin (*INS*) DNA. Circulating β cell-derived unmethylated *INS* DNA has a potential clinical value for the early detection of T1D, surveillance of islet transplantation rejection, and evaluation of response to therapy. Utilizing differentiated methylation patterns in different organs and employing a wide variety of molecular technologies also provide insights into the interrogation of biomarkers in other diseases with massive tissue-specific cell loss.

**Conclusion:**

Circulating unmethylated *INS* DNA is a promising molecular biomarker for the early detection of T1D.

## Background

Type 1 diabetes (T1D) is a complex disorder characterized by autoimmune destruction of β cell mass and hyperglycemia [1]. The loss of β cells may occur long before T1D can be diagnosed, given that T1D can only be diagnosed once approximately 65% of β cells have been killed [2, 3]. Therefore, early detection of β cell depletion may provide prompt therapeutic approaches or preventative interventions for type 1 diabetes mellitus.

Multiple genetic and environmental factors are involved in the onset of T1D. Therefore, a wide variety of biomarkers including the levels of insulin, proinsulin, and C-peptide [4], as well as circulating microRNAs [5] and HLA genotypes [6] have been investigated for the screening of high-risk individuals. However, none of these biomarkers has been effective, as they are only present after the insulin secretion ability has been significantly compromised, limiting their use in identifying ongoing β cell loss. Autoantibodies targeting antigens such as insulin; glutamic acid decarboxylase; protein phosphatase-like IA-2; zinc transporter-8 [7]; cytokines such as IL-1β, TNF, IFN-γ, and IFN family members; and T cell signatures [8], as well as the frequently used glucose levels, have been associated with β cell dysfunction [9]. These biomarkers of immune activation and β cell function can be used to evaluate the risk of developing T1D. However, they are limited in their ability to detect the loss of β cell mass and are more useful to monitor T1D progression [9]. Therefore, the lack of a method to directly detect β cell loss limits the possibility of detecting T1D within the potential therapeutic window period. Additionally, our understanding of the kinetics of T1D progression is limited because we do not have methods that directly measure the primary pathologic process, β cell death.

Promoter methylation controls tissue-specific gene expression. There has been an upsurge of interest in the exploration of circulating methylation markers as a diagnostic tool for cancer [10]. Extensively investigated biomarkers in this area include the *SEPT9* methylation blood test for colorectal cancer, which has been approved by the Food and Drug Administration (FDA) (http://www.accessdata.fda.gov/cdrh_docs/pdf13/p130001a.pdf). Progress in this field has inspired the development of novel methylation-based molecular biomarkers for other diseases with or without aberrations in DNA methylation patterns. This review summarizes recent findings in the development of β cell-derived circulating unmethylated insulin (*INS*) DNA for early detection of T1D.

## Potential of circulating unmethylated *INS* DNA as a diagnostic tool for T1D

**Fig. 1:**
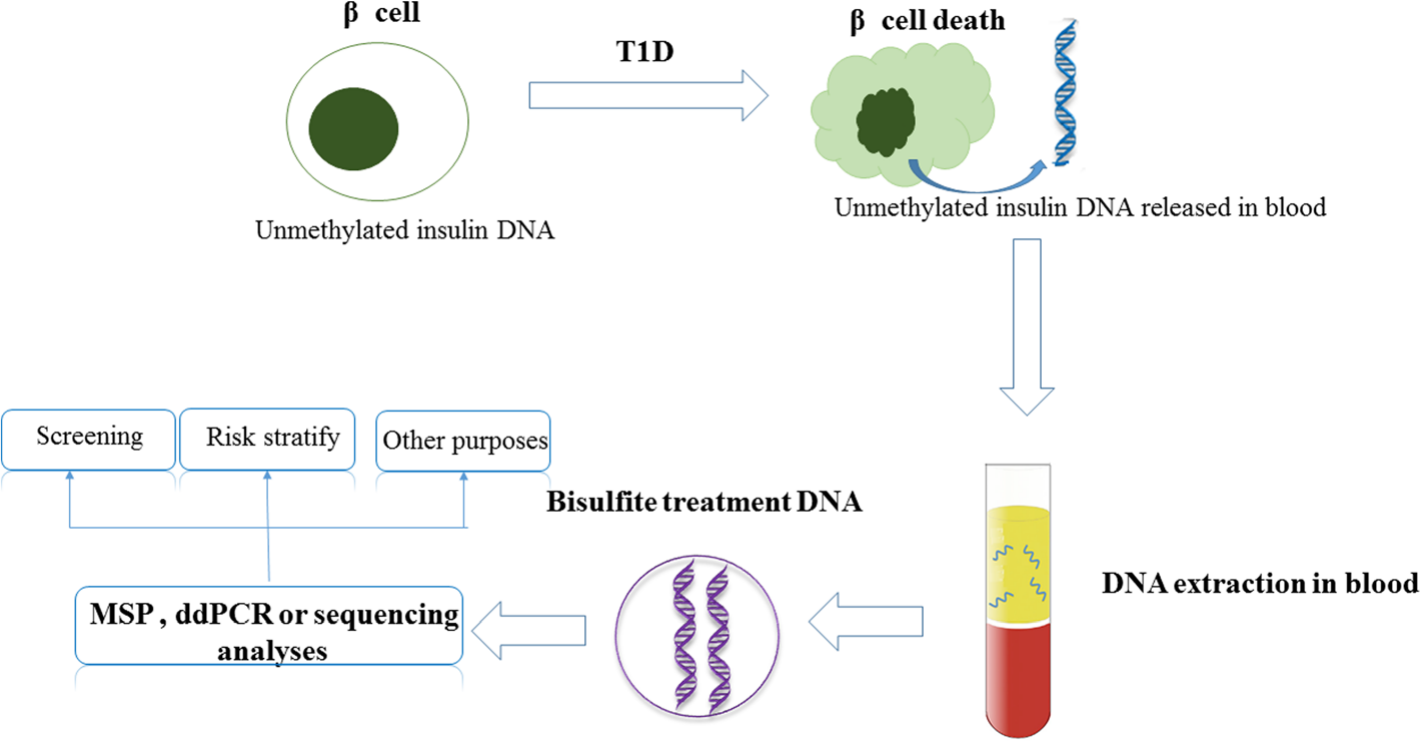
Circulating unmethylated *INS* DNA can be used to trace β cell death. The CpG sites of the *INS* gene are predominantly unmethylated in β cells, which is vastly different from those in other tissues. Autoimmune destruction conducted by immune cells can lead to direct damage of β cells. Unmethylated *INS* DNA is then released into the circulation. cfDNA can be extracted from blood samples. Following bisulfite treatment of cfDNA, unmethylated *INS* DNA molecules can be detected and quantified by a number of technologies including methylation-specific PCR(*MSP*), droplet digital PCR(*ddPCR*), and sequencing. Apart from the early diagnosis of T1D, the detection of circulating unmethylated *INS* DNA has the potential for monitoring transplantation rejection and response to therapy

Cell-free DNA (cfDNA) is a noninvasive “liquid biopsy” because changes in cfDNA can reflect physiological and pathological conditions [11]. Efforts have been made to identify tumor-specific cfDNA as reviewed by Schwarzenbach et al. [11]. Recently, the combination of cfDNA and cell-specific methylation patterns have provided a means by which β cell-specific circulating DNA can be detected. DNA methylation is an epigenetic event that modulates tissue-specific and developmentally regulated gene expression [12]. In this process, a methyl group is added to the 5′-position of cytosine of a CpG (cytosine-guanine) site. Tissue-specific methylation patterns make it possible to use differentially methylated DNA as a tool for diagnostic purposes. For example, the *INS* gene is uniquely unmethylated in the β cells of pancreatic islets [5, 13]. During the progression of T1D, β cells are destroyed by cytotoxic T lymphocytes; unmethylated *INS* DNA molecules are shed into the bloodstream and they become detectable (Fig. 1). This process allows the specific detection of β cell death in a minimally invasive manner, because the target sequences are specifically derived from β cells, not from other tissues such as blood cells, which contribute to the majority of cfDNA [11].

Based on the theory that each cell type in the body carries unique methylation marks, a number of studies have reported the specific methylation profile of insulin-secreting β cells in mouse or in human (Table 1) [13–16]. The most extensively investigated target of cell-specific DNA methylation is the *INS* gene. Due to the fact that the mouse genome consists of two insulin genes (*Ins1* and *Ins2*), both genes were examined for β cell-specific CpG sites [13, 14, 16]. Interestingly, four CpG sites in exon 2 of *Ins2* were revealed to be in a tissue-specific methylation pattern [14]. Comparing the levels of unmethylated DNA from islets with those from different tissues such as the lung, kidney, heart, blood, liver, brain, fat, and thymus, β cells were demonstrated to be the primary source of unmethylated *INS* DNA [13, 17, 18]. Sequencing of DNA from *INS*-positive β cells and *INS*-negative cells in mice revealed a 45-fold increase in unmethylated DNA in the insulin-positive cells [13], and the same trend was observed in human tissues [13, 14, 16]. The CpG sites were concentrated on the promoter region of the *INS* gene, and β cells showed an unmethylated *INS* status compared to cells from other tissues in human [15].

**Table 1:** Unmethylated loci in β cells identified by studies

Gene (source)	Position relative to the transcription start site	Reference
*Ins1* (mouse)	+177	13
*Ins2* (mouse)	+190, +310, +337, +340	14
*Ins2* (mouse)	−414, −182, −171	16
*INS* (human)	−357, −206, −135, −69, −19	15
*INS* (human)	−357, −345, −234, −206, −180, −135, −102, −69, −19	16

## Use of circulating unmethylated *INS* DNA to screen for T1D

The exploration of unmethylated CpG sites has promoted the development of methylation-specific primers or probes to assay β cell-released DNA molecules in the blood. Several groups have shown that circulating unmethylated *INS* DNA is a promising indicator of β cell death in both mouse models and humans. Akirav and colleagues [13] identified that circulating unmethylated DNA from β cells can be used to detect β cell death. In a mouse model in which β cells were acutely injured (BALB/c mice treated with high-dose streptozotocin), the demethylation index (2^(methylated cycle number) − (demethylated cycle number)^, representing the relative abundance of unmethylated DNA), was increased 2.6-fold and 3.8-fold 8 and 24 h after treatment, respectively. In non-obese diabetic (NOD) autoimmune mice, which simulate the chronic onset of T1D, the median demethylation index increased 21-fold prior to the decrease in insulin levels. In this study, a negative correlation was found between pancreatic insulin content and the demethylation index [13]. The results were consistent with those of other studies, in which significant elevated levels of circulating unmethylated *INS* DNA were detected well before the onset of hyperglycemia in high-/low-dose streptozotocin-administrated NOD/SCID mice and in NOD mice, respectively [14, 15, 17].

A higher level of unmethylated *INS* DNA was observed in five patients with new-onset T1D [13], and Lebastchi and colleagues further confirmed these results in a larger cohort study of 43 patients with new-onset T1D [19]. A follow-up study included a group of people with a “high risk” of developing T1D [20]. According to the study, relatives of T1D patients with two or more biochemical autoantibodies, abnormal glucose tolerance test results and normal HbA1c levels were considered as at-risk subjects [20]. This result revealed that the levels of unmethylated *INS* DNA were significantly higher in patients with recent T1D onset and at-risk individuals compared with those in non-diabetic controls [20]. The area under the receiver operating characteristic (ROC) curve was 0.834, allowing discrimination between T1D patients and health controls with a sensitivity of 38% and a specificity of 95%. The ability to identify at-risk subjects from non-diabetic control subjects was further enhanced, with the area of the ROC analysis being 0.897 with 58% sensitivity [20]. This was the first study to screen at-risk human subjects, the population that will benefit most from the early detection of T1D in a clinical setting. Furthermore, this was also the first time that ROC curve analyses were used to determine the diagnostic performance of unmethylated *INS* DNA in monitoring ongoing β cell death. A subsequent retrospective longitudinal study was conducted by the same group, in which an at-risk population was further divided into progressors (who developed T1D after a 3- to 4-year period) and nonprogressors (who did not develop T1D over a similar time interval) [21]. Comparison with healthy controls showed that progressors had a significantly increased unmethylation ratio (the levels of unmethylated *INS* DNA/the levels of methylated *INS* DNA). Furthermore, a strong inverse relationship between increased unmethylation ratio and ISR AUC (the area under the insulin secretion rate curve, representing the total insulin secreted in a 2-h test) was observed in progressors. More importantly, analysis of a separate group of “high-risk” individuals (shown by the presence of at least two autoantibodies and dysglycemia or impaired glucose tolerance) revealed that higher levels of unmethylated *INS* DNA can be measured, even when compared to the progressors and nonprogressors, indicating the potential of circulating unmethylated *INS* DNA to stratify the risk of T1D.

It is important to review several recently published results that present novel ideas for the investigation of circulating unmethylated *INS* DNA. Fisher et al. [22] determined the absolute levels of both unmethylated and methylated *INS* DNA in human using droplet digital PCR (ddPCR). Surprisingly, the levels of unmethylated *INS* DNA and methylated *INS* DNA were significantly increased at T1D onset. At 8 weeks post-onset, methylated *INS* DNA levels remained elevated while unmethylated *INS* DNA levels fell, and both parameters returned to control levels 1 year post-onset [22]. These observations have raised three questions: (1) How do the levels of both unmethylated and methylated *INS* DNA change in subjects with high risk of T1D prior to diagnosis? (2) How do the absolute levels of both parameters contribute to the demethylation index (as described in previous studies) to indicate the relative abundance of unmethylated DNA?; and (3) How does the autoimmune destruction of β cells, and other relevant cells, contribute to longitudinal changes in both parameters? Interestingly, during the progression of T1D in the NOD mouse model, the levels of methylated *INS* DNA in β cells can increase as a direct result of inflammatory destruction [23]. However, whether this increase contributes to the elevated levels of circulating methylated *INS* DNA requires further investigation.

Lehmann-Werman et al. [18] showed that the detection of several adjacent CpG sites could provide a better signal-to-noise ratio when measuring the levels of unmethylated circulating *INS* DNA. This concept is based on the dynamic nature of cells regulating tissue-specific methylation. In other words, the methylation status of a tissue may undergo stochastic changes due to aging (epigenetic drift), which can be enhanced by genetic and environmental factors [24–27]. This concept was consistent with previous results, showing methylated CpGs of the *INS* gene from β cells and unmethylated CpGs from other tissues [13–17]. By screening the combined methylation status of six CpG sites of the *INS* promoter (“unmethylated” or “methylated” defined by all six CpGs showing at least 80% similarity with the target sequences), the author significantly improved the sensitivity and the specificity of the assay. The results revealed that the levels of β cell-derived DNA were significantly higher in recently diagnosed T1D patients compared with those in health controls (10 vs. 175–1450 genomes/mL, *P* < 0.0001) [18].

## Major strengths of using methylation pattern to screen for T1D

The application of circulating *INS* DNA has a number of attractive features. First, the unique methylation pattern of the *INS* gene in β cells makes this approach specific and allows β cell destruction to be distinguished from the cell death of other tissues. This avoids the problems observed with cfDNA methylation-based biomarkers, whereby the aberrantly methylated loci in one malignancy type may also occur in other malignancies or diseases. Additionally, aberrant gene expression in the circulation may directly result from the effects of the tumor on blood cells, as white blood cells contribute the majority of cfDNA molecules [28]. Most importantly, DNA aberrations alone do not provide information pertaining to the exact source of these molecules.

Second, the use of blood for the assay is well suited for diagnostic purposes. As the most frequently explored starting material, obtaining blood can be steadily repeated and minimally invasive. This means that the assay is easily accessible to all subjects, including patients and at-risk individuals. Another major advantage is the inherent stability of cell-free DNA. Compared to other biomarkers such as microRNA and protein-based markers, DNA molecules are relatively stable. In the circulation, DNA molecules are predominantly found in the form of nucleosomes that either circulate as nucleoprotein complexes or adsorb to the surface of blood cells [29]. Furthermore, it is estimated that the half-life of unmethylated *INS* DNA is around 2 h [21], which means that the circulating levels of unmethylated *INS* DNA provide the opportunity to obtain real-time information of β cell mass loss in a noninvasive manner.

Finally, the measurement of circulating β cell-released DNA can be performed with a wide range of PCR-based molecular approaches, including methylation-specific real-time PCR [13–15, 17, 19], ddPCR [20–22], and sequencing [18]. The presence of tissue-specific methylation patterns makes it easier to develop methylation-specific methodologies. The rapid development of molecular technologies allows measurements of β cell-derived DNA to be identified using a small amount of starting material. For instance, a SYBR Green-based methylation-specific PCR can detect as few as 10 copies of unmethylated *INS* DNA circulating within genomic DNA with a coefficient of variation from 21.64 to 38.72% [14, 15]. Furthermore, the use of TaqMan PCR enhanced the discriminatory capability of methylation-specific PCR with relatively high signal-to-noise ratio, linear assay output, and simultaneous detection of methylated and unmethylated *INS* DNA in a single PCR mix [17]. It is of note that ddPCR, which enables direct quantitation of differentially methylated DNA species in serum without the need for normalization, has been widely used in the research of β cell-released DNA, one of which obtained 1.87 and 1.37% as the coefficients of variation from two sample sets [20–22] The multiplexed ddPCR developed by Usmani-Brown et al. allowed the detection of about 0.7 copies/μL of unmethylated *INS* DNA targets [20]. Furthermore, Lehmann-Werman et al. [18] used sequencing to identify unmethylated *INS* DNA with remarkably enhanced sensitivity and specificity, as well as low cost (about $10 each sample), making the simultaneous detection of several adjacent CpGs a reality. We believe that sequencing technologies will be increasingly important in upcoming studies.

## Future prospects

In addition to the early detection of T1D and screening of at-risk subjects, circulating unmethylated DNA could be of clinical value for other purposes such as monitoring transplantation rejection and response to therapy. In islet transplantation cases, demethylated *INS* DNA could be detected from day 1 to day 14 post-transplantation [15]. Another study, focusing on a shorter period of time after transplantation, found that β cell death peaked by 360 min post-transplantation in four autologous islet recipients [21]. Lebastchi et al. [19] provided additional insight into the effects of medication on β cell death. Compared to placebo-treated controls, a dramatic decline in the relative level of unmethylated *INS* DNA (Ct value of methylated DNA − Ct value of unmethylated DNA) was observed in patients after 1 year of treatment with teplizumab, indicative of a reduced level of β cell destruction as a result of immune therapy [19].

A recent report described the use of the amylin gene, which is highly expressed in β cells with unique unmethylated patterns in the coding region, in the identification of β cell loss [30]. The ROC analysis showed an AUC of 0.866 to discriminate subjects with recent onset of T1D from healthy controls [30]. These results raise the possibility that there are other genes harboring β cell-specific methylation patterns. We believe that with technological advances, more target genes of interest can be explored and readily combined into panels to optimize the sensitivity and specificity of these assays. Another recent study reported a negative relation between the onset of gestational diabetes mellitus (GDM) and β cell loss by using the detection of unmethylated *INS* DNA [31]. Interestingly, the levels of unmethylated *INS* DNA were significantly decreased in women with GDM comparing to women with normal pregnancy, women at postpartum, and non-pregnant women [31]. The study told us that the detection of unmethylated *INS* DNA may provide more detailed information about the natural history and heterogeneity of T1D, which is crucial to illustrate the etiology of T1D [32].

The small proportion of β cell-released DNA within the background of circulating genomic DNA poses a technical challenge. Recently developed high-throughput molecular techniques with improved accuracy, such as multiplex ddPCR and next-generation sequencing (NGS), are anticipated to bring major improvements in this field. The ability to accurately perform low-level absolute quantification without the need of calibration makes ddPCR a popular technology for molecular diagnostics [33], while multiplex ddPCR enables the simultaneous quantification of more than five targets [34]. Sequencing has been applied in ongoing studies to overcome the issue of conventional methylation-specific PCR, which has high background signals resulting from nonprogrammed methylation, or demethylation, of CpG sites [18]. Using Illumina Miseq sequencing, the methylation status of four to nine CpG sites can be defined in a single reaction [18]. Another novel approach is genome-wide bisulfite sequencing. Using this approach, Sun et al. [28] produced a “tissue map” by identifying the relative proportions of DNA released from multiple tissue origins in the plasma of pregnant women, transplant recipients, and patients suffering from hepatocellular carcinoma. Such approaches will facilitate research into, and the development of, genome-wide tissue-specific methylation profiles. Furthermore, these studies are likely to yield advances in the development of footprints for a wide variety of cell types with specific epigenetic signatures [35, 36] and in other areas including finding the origin of forensic samples [37] and archeology researches [38].

## Conclusions

The studies discussed above provided evidence that circulating unmethylated *INS* DNA can be a potential noninvasive biomarker of β cell mass loss in T1D. Besides the early detection, this approach may also aid risk stratification, disease surveillance, and treatment response assessment in T1D. The feasibility of serial sampling makes the generation of a dynamic profile of β cell mass destruction a reality. In addition, these studies offer new insights for the generation of novel biomarkers based on tissue-specific methylation patterns in cfDNA.

On the other hand, analyses of β cell-derived unmethylated DNA in T1D are still in the early stages. A review of these studies reveals two major limitations that will need to be addressed in the coming years. One limitation is the relatively small sample size used in these studies, yet prospective and screening assessments for the diagnostic performance of circulating *INS* DNA remain warranted. Another concern is the inconsistent results obtained in the studies. The reasons for inconsistent results could be heterogeneous, including the use of different techniques with different sensitivities and differences in the target populations of study. These discrepancies can be addressed through the development of standardized processing protocols, which would allow comparisons between groups.

## Abbreviations

cfDNA: Cell-free DNA
ddPCR: Droplet digital PCR
*INS*: Insulin
ROC: Receiver operating characteristic
T1D: Type 1 diabetes
